# Modelling improved efficiency in healthcare referral systems for the urban poor using a geo-referenced health facility data: the case of Sylhet City Corporation, Bangladesh

**DOI:** 10.1186/s12889-020-09594-5

**Published:** 2020-09-29

**Authors:** Alayne M. Adams, Rushdia Ahmed, Shakil Ahmed, Sifat Shahana Yusuf, Rubana Islam, Ruman M. Zakaria Salam, Rocco Panciera

**Affiliations:** 1 Department of Family Medicine, Faculty of Medicine and Health Sciences, McGill University, 5858 Cote des Neiges, Room 332, Montréal, H3S 1Z1 Québec, Canada; 2 Health Systems and Population Studies Division, icddr,b, Dhaka, Bangladesh; 3 School of Public Health & Community Medicine, University of New South Wales, Sydney, Australia; 4 Implementation Research and Delivery Science Unit, Health Section, UNICEF, New York, NY USA

**Keywords:** Referral system, Referral mechanism, Referral inefficiencies, GIS, Health systems, Urban, Bangladesh

## Abstract

**Background:**

An effective referral system is critical to ensuring access to appropriate and timely healthcare services. In pluralistic healthcare systems such as Bangladesh, referral inefficiencies due to distance, diversion to inappropriate facilities and unsuitable hours of service are common, particularly for the urban poor. This study explores the reported referral networks of urban facilities and models alternative scenarios that increase referral efficiency in terms of distance and service hours.

**Methods:**

Road network and geo-referenced facility census data from Sylhet City Corporation were used to examine referral linkages between public, private and NGO facilities for maternal and emergency/critical care services, respectively. Geographic distances were calculated using ArcGIS Network Analyst extension through a “distance matrix” which was imported into a relational database. For each reported referral linkage, an alternative referral destination was identified that provided the same service at a closer distance as indicated by facility geo-location and distance analysis. Independent sample t-tests with unequal variances were performed to analyze differences in distance for each alternate scenario modelled.

**Results:**

The large majority of reported referrals were received by public facilities. Taking into account distance, cost and hours of service, alternative scenarios for emergency services can augment referral efficiencies by 1.5–1.9 km (*p* < 0.05) compared to 2.5–2.7 km in the current scenario. For maternal health services, modeled alternate referrals enabled greater referral efficiency if directed to private and NGO-managed facilities, while still ensuring availability after working-hours. These referral alternatives also decreased the burden on Sylhet City’s major public tertiary hospital, where most referrals were directed. Nevertheless, associated costs may be disadvantageous for the urban poor.

**Conclusions:**

For both maternal and emergency/critical care services, significant distance reductions can be achieved for public, NGO and private facilities that avert burden on Sylhet City’s largest public tertiary hospital. GIS-informed analyses can help strengthen coordination between service providers and contribute to more effective and equitable referral systems in Bangladesh and similar countries.

## Background

With projections indicating that 68% of the world’s population will live in urban areas by 2050 [1], rapid urban growth has been identified as one of the major threats to health in the twenty-first century [2]. Even though urbanization brings economic and social progress, in low- and middle-income countries (LMICs) it is accompanied by a variety of health challenges related to inadequate housing, poverty, overburdened infrastructure, toxic air quality, and traffic congestion [3]. Occurring simultaneously is a burgeoning healthcare market dominated by the private sector [4, 5].

In Bangladesh, the largely unregulated private sector accounts for approximately 90% of health facilities in urban areas [6], with attendant consequences for cost, equity of access and quality of care provided [7–9]. Navigating this complex pluralistic healthcare landscape in a manner that ensures timely access to appropriate and affordable services is not straightforward, nor is there an organized referral system in place to assist [10]. For the urban poor who rely on the largely informal private sector as a first point of care such as drug shops or consultation chambers [8, 11], reports of erroneous referrals, delays in treatment and or impoverishing additional or unnecessary costs of care are common [12]. Referral inefficiencies have also been linked to excessive distance from facilities, unsuitable hours of operation, by-passing lower level hospitals and the inappropriate diversion of patients by middle men or *dalals* [7, 12–14].

Efficient referral systems are a fundamental aspect of primary healthcare delivery [15, 16], enabling access to timely, appropriate, and affordable health services. Referral is defined as “a process in which a health worker at one level of the health system, lacking sufficient resources (drugs, equipment, skills) to manage a clinical condition, seeks the assistance of a better or differently resourced facility at the same or higher level to assist in, or take over the management of, the client’s case” [17]. In emergent situations like obstetric complications or cardiac arrest, appropriate referral systems that take into consideration service quality, distance, and facility infrastructure, are particularly important in ensuring patient survival and well-being [18–20]. An organized referral chain also assists in making cost-effective use of hospitals and primary healthcare services [17].

According to the literature, referral systems that formalize communication and transport arrangements between different healthcare facility levels are especially important [18, 21, 22]. Indeed, the effectiveness of health delivery depends substantially on the efficiency of referral systems in minimizing delays in needed treatment by reducing travel time, identifying the closest appropriate provider [19, 22] and supporting optimal utilization of the health workforce [18, 20, 23]. For this reason, characterizing gaps in efficient referrals [24] and designing structured referral systems sensitive to patient needs and appropriate quality care [16] should be prioritized.

An efficient referral system is especially crucial in the context of pluralistic health systems like Bangladesh, enabling a more systematic means of ensuring appropriate, timely, and affordable healthcare is available and accessible to the population [7–9, 21]. Of particular concern are inefficiencies in referral from the primary contacts utilized by the urban poor (drug shops, traditional healers and consultation chambers etc.) to appropriate qualified secondary and tertiary providers [7, 12].

While referral systems are traditionally analysed from a patient-oriented, diagnostic process perspective [16, 23–28], in efforts to address inefficiencies in urban settings characterized by dense service provision and traffic, there is also merit in considering the geospatial dimension of referral. To date, several studies have investigated the use of Geographic Information Systems (GIS) to inform decision-making about interventions to increase access to emergency services [19, 21, 22, 29, 30]. In this paper, we explore the potential of GIS-informed modeling to support the development of referral networks for the urban poor that consider the geographic proximity of referral facilities, as well as service quality, hours of operation and costs. Of particular interest is how different actors within a pluralistic health system (e.g., private, public, NGO) interact within reported referral networks, and whether efficiencies in this network can be improved. This analysis is particularly timely given that Bangladesh’s 2016–2021 Health, Nutrition and Population Strategic Investment Plan identifies an efficient referral system as a critical priority in ensuring a close relationship between all levels of the health system, and enabling people’s access to the best possible care closest to home [31].

## Methods

This quantitative modeling exercise uses data from a geo-referenced census of public, private and NGO health facilities in Sylhet City Corporation (SCC), Bangladesh that included information on reported referral practices. Conducted from November 2012 to February 2013, the SCC census was part of a larger urban mapping project undertaken by icddr,b in major cities and municipalities across the country [32]. SCC is the capital of Sylhet district that ranks among the more economically advantaged areas of Bangladesh, yet is the poorest performing on a range of health indicators including infant and child mortality, and maternal and newborn health [33].

### Data collection and processing

Prior to the facility census, four GIS-trained staff conducted “ground truthing” to update base maps constructed using municipal road network data and Google images. The SCC base maps were collected from *Urban Partnerships for Poverty Reduction* (UPPR) initiative supported by Government of Bangladesh, UKaid and UNDP Bangladesh [34]. Facilities were subsequently listed, geo-referenced and surveyed ward-by-ward by seven teams of trained field staff using a hand-held tablet computer loaded with updated base maps. This process was conducted over several stages to ensure that no facility was overlooked. A team of data managers compiled digital data from the field on a daily basis, then shared it with team leaders supervising data consistency and quality. If any anomalies were detected, these were immediately addressed through additional field visits. In addition, three staff were assigned to make randomly selected spot checks to verify that all geo-referenced facilities were surveyed.

### Facility information

The facility census collected information on the type of facility, services offered, hours of operation, staff qualifications, referral practices, and cost for specific services. Data were collected from the most informed person at each facility, which in the majority of cases was the owner or manager. Multiple visits were often required. Neither the veracity of reported data, nor the quality of services offered was determined.

The census dataset for SCC contained a total of 1251 health facilities. Of these, the majority (47.6%) were drug shops and pharmacies, private doctors’ consultation chambers attached to pharmacies, and standalone doctor’s chambers. Table 1 depicts their distribution according to type of facility: public, private or NGOs. Within the public category is the teaching hospital, Sylhet MAG Osmani Medical College & Hospital (here onward referred to as Osmani Medical College), which is the largest tertiary level facility in the district. The NGO category is largely comprised of donor-funded and NGO-managed primary care facilities targeting the urban poor, either working independently or contracted-out by local government. The latter group was not categorized into the public category given its dependence on external funding. Finally, while all facilities reporting referral were considered in this analysis, only “formal” facilities with MBBS qualified doctor(s) on staff were considered candidates for redirected referral.

**Table 1:** Distribution of health facilities in Sylhet City Corporation reporting referral by management entity

Type	Public	NGO^a^	Private	Total
Hospital (in and outpatient services > 30 beds)	6	0	16	22
Clinic (in and/or outpatient services < 30 beds)	1	67	53	121
Diagnostic centre (mainly medical testing & imaging)	0	0	49	49
Doctor’s chambers (independent private practice)	0	0	151	151
Pharmacy attached with Doctor’s chamber	0	0	168	168
Drug shops & pharmacy (sell drugs for profit, rarely with licensed pharmacist)	0	0	596	596
Delivery huts (pregnancy & delivery care services by NGO trained CHWs, mid-wives or birth attendants)	0	15	0	15
EPI centres (immunization services offered periodically)	83	4	0	87
**Total**	**90**	**86**	**1033**	**1209**

^a^42 NGOs in this category are contracted out by local government as part of the Urban Primary Health Care Project (UPHCP), funded by the Asian Development Bank

### Reported referral linkages

Information on “referral linkages” was solicited from owners or managers of each facility in the census. Specifically, respondents were asked to indicate the services for which patients were typically referred elsewhere, and for each of these services, the name of the facility where patients were typically referred. Solicited in this manner, reported referral arrangements were not based on actual patient-specific referral events, nor were they verified with the receiving facility. For this reason, they are heretofore referred to as ‘reported referral linkages’, while the “referral network” is the ensemble of referral linkages reported at a given facility. Each referral linkage is analyzed in terms of the “originating” facility and the reported “receiving” facility for a particular health service.

### Service groups

The facility database for SCC provides information on the specific services that each health facility provides. For some of these services (e.g. urine sugar and skin disease), the efficiency of a referral network is not as crucial as for others. For this reason, this study focused on two groups of services for which timely referral is particularly important: (i) emergency and critical care services such as trauma, burn injury, heart disease, stroke, as well as facilities like Intensive Care Unit (ICU), Newborn Intensive Care Unit (NICU) and Coronary Care Unit (CCU), and (ii) Maternal health services including Emergency Obstetric Care (EmOC), Normal Vaginal Delivery (NVD), and Caesarian Section delivery (C-section).

### Calculation of geographic distances

By identifying originating and receiving facilities using facility names and geo-locations, a geographic distance (in km) was calculated for each reported referral linkage using ArcGIS Network Analyst extension. In this manner, a “distance matrix” was created for each reported referral linkage. This distance matrix was imported into a relational database containing facility survey data, thus enabling a distance-based analysis of referrals. Travel time is generally modelled from geographic distances based on assumptions about transit speeds [35–37]. Transit speed is associated with a variety of dynamic factors (individual-level choice and availability of transport, highly variable traffic conditions). Modelling travel time in a way that accurately reflects the variety of possible situations required for this analysis is challenging due to difficulties in obtaining and processing detailed information on how transit speed changes spatially and for different individuals. For SCC, the only information available on traffic conditions and transport was collected at a few major intersections [38]. Given the absence of more accurate information on spatial variability of transit speeds, the decision was made to express the results of the referral analysis in terms of distance as this is an absolute, well-measured factor. This should have no impact on the conclusions reached in terms of referral efficiency. Because we modelled travel time under the traditional assumption of constant transit speed across the network, a difference in distance between referral options will likely result in a proportional relative difference in travel time.

### Data analysis

Analysis consisted of two steps. First, the referral network was analyzed in terms of linkages between different management entities (formal public, private for-profit and NGOs). This was done to shed light on the referral relationships between the main healthcare actors in Bangladesh’s urban healthcare system. The distance between the originating and the receiving facility, the cost of services at the receiving facility, the availability of qualified MBBS trained doctors, and the opening hours of the receiving facility were also considered. Opening hours are considered a parameter of accessibility to healthcare by the urban poor given evidence that shows the propensity of the working poor to seek care during evening hours [7, 8].

In a second step, alternate referral scenarios were developed to assess the extent to which the existing referral system might be improved to better serve the urban poor. For each existing reported referral linkage, an alternative referral destination was identified that provided the same service requested by the originating facility but was closer in terms of road network distance. The availability of qualified personnel i.e. MBBS doctors at each receiving facility and 24 h of operation were also considered in determining alternate referral destinations as were service costs. Although comprehensive data were not available for every facility surveyed, average costs of normal and caesarean delivery and emergency and critical care were calculated for each management entity (see Table 2). Despite our focus on affordable healthcare for the urban poor, we retained private facilities in our analysis of alternate referral networks given their substantial presence in urban areas, availability during evening hours, and important contribution to the delivery of critical care services (i.e. ICU, CCU, NICU) beyond what is supplied by the already oversubscribed public tertiary hospital. Independent/unpaired sample t-tests with unequal variances were performed to analyze the differences in distance comparing the reported referral link and the alternate referral scenario. A level of significance of 0.05 was used for all analyses.

**Table 2:** Estimated official cost information for facility delivery and emergency services by management entity

Cost of services	Public		NGO		Private	
	Mean^a^	Median^a^	Mean	Median	Mean	Median
**Delivery services**						
C-section single	7.5	7.5	8000	8000	11,250	13,250
Normal vaginal delivery single	7.5	7.5	525	500	6660	6000
C-section package^b^	–	–	11,500	12,500	19,620	21,000
Normal vaginal delivery package^b^	–	–	3850	4000	6222	7000
***Approximate delivery cost***	***7.5***	***7.5***	***5637***	***4500***	***12,650***	***8100***
Emergency services						
Intensive Care Unit (ICU)	10	10	–	–	1974	2000
Coronary Care Unit (CCU)	10	10	–	–	2271	1600
Newborn Intensive Care unit (NICU)	–	–	–	–	3333	3000
***Approximate emergency cost***	***10***	***10***	***–***	***–***	***2337***	***2000***

^a^These are official costs in Bangladesh taka, and do not consider informal payments charged at public facilities which can sometimes be substantial [39, 40]

^b^Package means bundled services and products together at a fixed price for conducting a medical procedure such as normal vaginal delivery or C-section. Such packages are negotiated between the facilities and patients and could include hospital stay, operation theatre charge, and other related costs along with the cost of the procedure itself [12]

### Ethical considerations

The protocol for this project (PR-13100) was approved by both Research and Ethical Review Committees of the icddr,b, Dhaka, Bangladesh. Permission was sought from the appropriate authority prior to facility census and survey. For public facilities, this involved obtaining signatures from officials at the Ministry of Health or Local Government, and from the relevant administrative authority in not-for-profit facilities. For smaller private sector clinics, signed consent was obtained from the person responsible at each health facility before health service information was requested. Participation was completely voluntary, and efforts were made to collect service information at the respondent’s convenience to minimize disruption of normal business activities.

## Results

Among a total of 670 reported referral linkages in the database, 339 concerned emergency and critical care services related to trauma, heart disease and stroke.

Table 2 displays the approximate cost of facility delivery and selected emergency services in public, private, and NGO managed facilities. As expected, and consistent with previous research, service costs in public facilities were substantially lower than both NGO and private facilities, NGO services were approximately half the cost of those in private facilities and the largest cost variations occurred in formal private sector facilities [41]. There is substantial evidence that private sector services are accessed by the urban poor due to their dominant presence in urban areas, despite the risk of medical impoverishment [8, 12, 42].

In Tables 3 and 4, reported referral linkages for emergency care and maternal health were compared to an alternate referral scenario in which referrals are redirected to a closer facility providing similar services. As noted previously, alternative referral scenarios include the private sector given its important contribution to overall reported referral linkages. The relative service cost of both alternative and referral scenarios is implied in the choice of management entity, whereby the costs assumed by patients presenting at public or NGO facilities are substantially lower than the private sector. Hours of service are also indicated in each of the alternative referral scenarios. Using the example of heart disease in Table 3, a total of 163 referrals were reported for these services at an average distance of 2.6 km. Among these referrals, 82% or 134 were towards public health facilities involving an average distance of 2.7 km. However, in the alternative scenario, 83% (135 referrals) could be redirected towards to closer public facilities averaging 1.8 km from the referring facility. As noted in the last column, this difference is highly significant.

**Table 3:** Current reported referral linkages for emergency and critical care services in Sylhet City Corporation compared to an alternate referral scenario in which the receiving facility provides the required service but at a closer distance^a^

Total # of reported referrals & their average distance	Current scenario - % of total reported referrals received, their average distance & evening availability	Alternate scenario - % of total reported referrals that could be redirected, & their average distance	***p*** value
Heart Disease 163 @ 2.6 km	82% to Public @ 2.6 km (100% evn)	83% to Public @ 1.8 km (100% evn)	0.0000**
	2% to NGO @ 3.5 km (0% evn)	0% to NGO	–
	16% to Pvt. @ 1.6 km (100% evn)	66% to Pvt. @ 0.6 km (95% evn)	0.0000**
Stroke 72 @ 2.4 km	82% to Public @ 2.5 km (100% evn)	85% to Public @ 1.9 km (100% evn)	0.0058**
	0% to NGO	0% to NGO	–
	18% to Pvt. @ 2.2 km (100% evn)	79% to Pvt. @ 0.4 km (100% evn)	0.0009**
Major Trauma 104 @ 2.5 km	93% to Public @ 2.5 km (100% evn)	83% to Public @ 1.5 km (100% evn)	0.0000**
	1% to NGO @ 1.8 km	0% to NGO	–
	6% to Pvt. @ 1.6 km (100% evn)	90% to Pvt. @ 0.9 km (100% evn)	0.0324**
Burn Injury 139 @ 2.7 km	91% to Public @ 2.7 km (100% evn)	86% to Public @ 1.8 km (100% evn)	0.0000**
	0% to NGO	0% to NGO	–
	9% to Pvt. @ 2.1 km (100% evn)	90% to Pvt. @ 0.9 km (100% evn)	0.0002**
ICU 16 @ 2.9 km	75% to Public @ 2.9 km (100% evn)	19% to Public @ 2.2 km (100% evn)	0.2456
	0% to NGO	0% to NGO	–
	25% to Pvt. @ 3.1 km (100% evn)	100% to Pvt. @ 0.7 km (100% evn)	0.0165**
CCU 13 @ 3 km	85% to Public @ 2.9 km (100% evn)	8% to Public @ 3.7 km(100% evn)	–
	0% to NGO	0% to NGO	–
	15% to Pvt. @ 3.9 km (100% evn)	100% to Pvt. @ 0.7 km (100% evn)	0.1091
NICU 12 @ 3 km	83% to Public @ 2.9 km (100% evn)	0% to Public	–
	0% to NGO	0% to NGO	–
	17% to Pvt. @ 3.9 km (100% evn)	100% to Pvt. @ 1.3 km (100% evn)	0.1297

Public = Government, Pvt. = Private, evn = evening ^a^Distances represent the average distance amongst all referrals in each group, and percentages are calculated based on total originating referrals (column 1) **p < 0.05

**Table 4:** Current reported referral linkages for maternal health services in Sylhet City Corporation compared to an alternate referral scenario in which the receiving facility provides the required service but at a closer distance^a^

Total # of reported referrals & their average distance	Current scenario - % of total reported referrals received, their average distance & evening availability	Alternate scenario - % of total reported referrals that could be redirected, & their average distance	***P*** value
EMOC 44 @ 2.6 km	70% to Public @ 2.7 km (100% evn)	68% to Public @ 1.8 km (100% evn)	0.0076**
	16% to NGO @ 2.1 km (86% evn)	73% to NGO @ 1.4 km (100% evn)	0.0076**
	14% to Pvt. @ 2.4 km (100% evn)	82% to Pvt. @ 1.2 km (100% evn)	0.0568
NVD 54 @ 2.8 km	61% to Public @ 2.9 km (100% evn)	71% to Public @ 1.9 km (100% evn)	0.0012**
	32% to NGO @ 2.6 km (76% evn)	91% to NGO @ 0.9 km (88% evn)	0.0000**
	7% to Pvt. @ 2.4 km (100% evn)	98% to Pvt. @ 0.8 km (100% evn)	0.1311
C-Section 53 @ 2.4 km	64% to Public @ 2.5 km (100% evn)	72% to Public @ 1.7 km (100% evn)	0.0082**
	21% to NGO @ 2.7 km (73% evn)	72% to NGO @ 1.4 km (71% evn)	0.0052**
	15% to Pvt. @ 1.4 km (100% evn)	94% to Pvt. @ 0.6 km (100% evn)	0.0431**

Pvt. = Private, evn = evening ^a^Distances represent the average distance amongst all referrals in each group, and percentages are calculated based on total originating referrals (column 1) **p < 0.05

For all services, irrespective of emergency care or maternal healthcare, the majority of reported referral linkages were received by public facilities and to a much lesser extent, by other private facilities. Column 2 of Table 3 indicates reported referral linkages for conditions falling into the emergency and critical care services group. Those received by public facilities involve average distances between 2.5–2.9 km, and the very few reported referral linkages received by NGOs have average distances between 1.8–3.5 km. Average distances involved in reported referral linkages received by private facilities were shorter (1.4–2.2 km) for all services except CCU, ICU and NICU (2.9 km for public vs 3.1–3.9 km) compared to public facilities, perhaps reflecting the greater availability of private facilities in the municipality. It is noteworthy that no referrals for emergency services were directed to NGOs.

In terms of reported referral linkages for maternal health services (see column 2 Table 4), more than 60% of referrals were made to public facilities at an average distance of 2.5 km to 2.9 km. A smaller proportion were referred to NGO facilities (16–32%) at an average distance of 2.1–2.7 km distance, not all of which had evening services available. Even though private facilities offer evening maternal health services, they were reported to receive only 15% of referrals at an average distance of 1.4–2.4 kms.

Overall, alternate scenarios modeled in column 3 of Tables 3 and 4 demonstrate substantial reductions in referral distance if directed to public facilities other than Osmani Medical College (the main tertiary teaching hospital in SCC) which offer the same services but at closer distances. Table 5 provides a service-specific view of the extent to which reported referral linkages focus on Osmani Medical College and indicates alternative referrals to closer public hospitals which offer the same services.

**Table 5:** Percentage of reported referral linkages for all type of services to Osmani Medical College Hospital vs. other public hospitals

Services	Facilities in Sylhet City Corporation	Current (%)	Alternative (%)
EmOC	Osmani Medical College Hospital	97	3
	Other public hospitals	3	97
NVD	Osmani Medical College Hospital	97	0
	Other public hospitals	3	100
C-Section	Osmani Medical College Hospital	97	0
	Other public hospitals	3	100
Heart Disease	Osmani Medical College Hospital	99	1
	Other public hospitals	1	99
Stroke	Osmani Medical College Hospital	98	0
	Other public hospitals	2	100
Major Trauma	Osmani Medical College Hospital	99	0
	Other public hospitals	1	100
Burn Injury	Osmani Medical College Hospital	100	0
	Other public hospitals	0	100
ICU	Osmani Medical College Hospital	100	100
	Other public hospitals	0	0
CCU	Osmani Medical College Hospital	100	100
	Other public hospitals	0	0
NICU	Osmani Medical College Hospital	100	–
	Other public hospitals	0	–

**Fig. 1:**
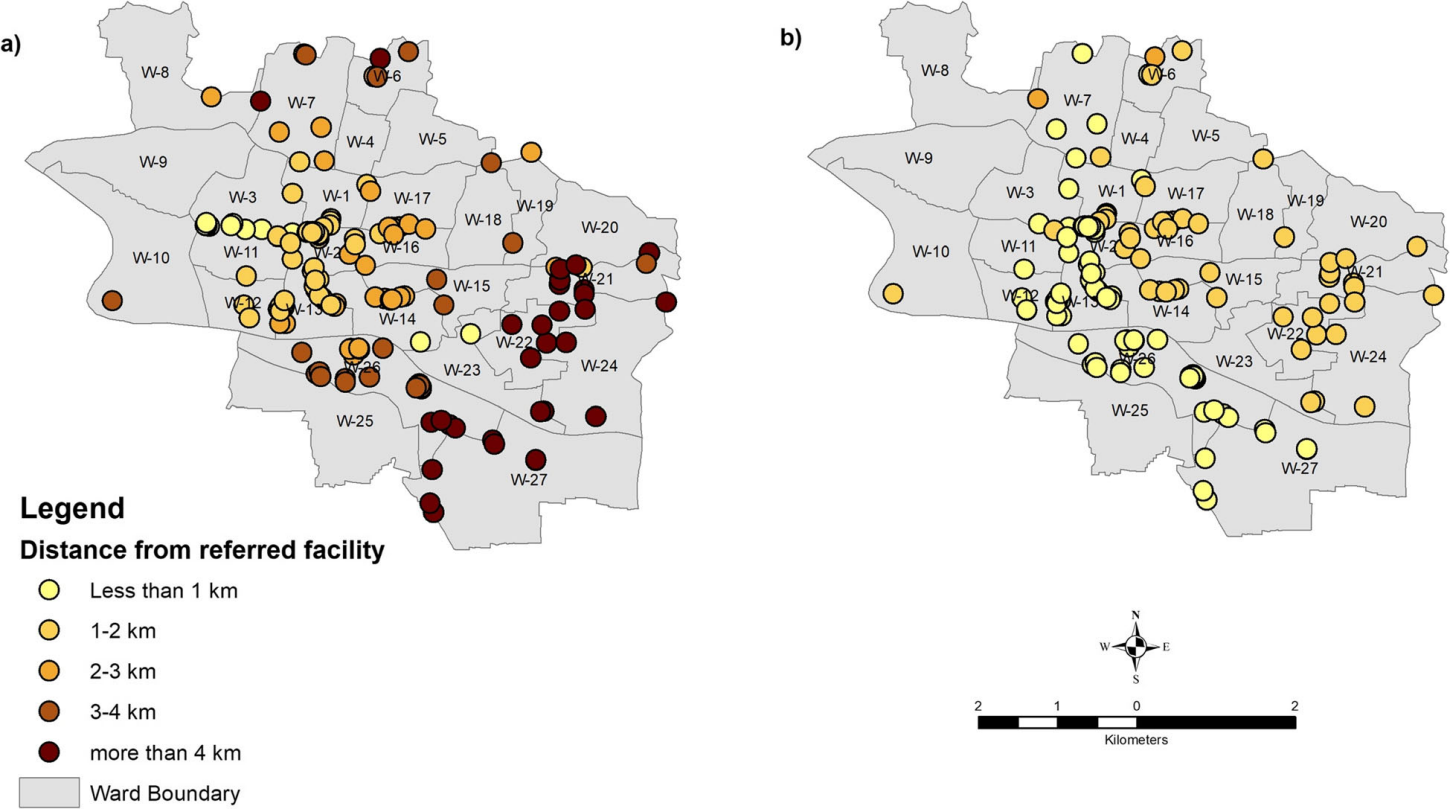
Distance for heart disease treatment from the referral destination to: **a**) the current reported referral destination, and **b**) the alternative, closer and appropriate, referral destination

For emergency and critical care services (Table 3), the maximum referral distance in the alternative scenario is 2.2 km, against 3.9 km in the current scenario. More specifically, distances associated with reported private sector referrals for heart disease, stroke, trauma and injury which are largely directed to Osmani Medical College, could be significantly reduced if closer alternative public and private hospitals were used. For heart disease, stroke, trauma and burn injuries, between 83 and 86% of reported referrals could be received by closer public facilities at average distances of 1.5 to 1.9 km (*p* < 0.05) compared to 2.5 to 2.7 km in the current scenario. Similarly, while the current scenario indicates that the majority of cases requiring ICU, CCU, and NICU care were reportedly referred to public facilities (75–85%) at an average distance of 2.9 km, the alternative scenario proposes referrals to the private sector that would reduce distances to between 0.7–1.3 km. To geospatially represent these efficiencies, Fig. 1 maps the reduction in distance for referral for heart disease when instead of current reported referral destinations, patients are redirected to the nearest facility with qualified providers providing similar services. It is clearly evident that the distance to destination facilities are noticeably reduced in alternative scenario (Fig. 1b) for the facilities in the eastern and southern part of the city.

As shown in Table 4, for maternal healthcare services, approximately 70% of reported referrals could be received by 3 other public facilities at significantly reduced average distances of 1.7 to 1.9 km (*p* < 0.05) compared to current distances of 2.5 to 2.9 km. The alternate scenario (column 3) further suggests that NGOs could receive a significant portion (72 to 93%) of current reported referral linkages at significantly reduced average distances of 0.9 to 1.4 km (*p* < 0.05) compared to average distances of 2.1 to 2.7 km in the current scenario.

Overall, the maximum referral distance for emergency and critical care services in the alternative scenario is 2.2 km, against 3.9 km in the current scenario if closer public or private sector facilities are used. Similarly, for maternal healthcare services the alternative referral scenario proposes maximum distances of 1.9 km against 2.9 km in the current scenario but involves a higher utilization of NGOs as referral destinations.

It should be noted, however, that a greater reliance on private sector referrals may not be ideal for the urban poor due to the substantial costs of care associated with these facilities. Nevertheless, the alternative scenario for emergency and critical care services guarantees that the receiving facility will be open after working hours (with the exception of heart disease), which is a more convenient window for seeking care among the working poor. This is also true for maternal healthcare services referrals (NVD and C-Section), redirected to private facilities.

## Discussion

In this paper we explore the potential of GIS-informed modeling to increase efficiencies in facility-based referral practices based on considerations of proximity, quality, hours of operation and cost with a particular focus on the urban poor. Using the example of Sylhet City Corporation in Bangladesh, we note a pluralistic healthcare system involving public, private and NGO healthcare actors and patterns of referral that fail to take efficiency into account. A first observation arising from the assessment of current reported referral networks in Sylhet City concerns the crucial importance of its large public hospital, Osmani Medical College as a referral destination. The substantial number of facilities reporting referrals to Osmani Medical College across all services (both emergency and maternal care) is indicative of the population’s dependence on low cost public facilities irrespective of the greater distances involved. This is not without dangers, however, especially in the context of emergencies when proximity of care may be critical to survival. Many of the reported emergency-related referrals to Osmani Medical College originate from facilities that are over 2.5 km distant.

Overcrowding at Osmani Medical College, with a reported bed occupancy rate of 190%, is a further concern [43] and indicates a lack of capacity to deal with patient volume. Problems with overcrowding at the tertiary level are not unique to Bangladesh. In many low-income countries, healthcare utilization studies indicate that large numbers of patients bypass primary healthcare facilities in favor of tertiary level facilities despite substantial additional time and financial costs [17, 44]. The development of formalized referral systems that direct patients to appropriate levels of care at primary or secondary facilities could therefore reduce the costs and burden of using highly skilled human resources at the tertiary level.

The modeling exercise conducted in this paper suggests that greater efficiency in referral is possible. When reported referral linkages for maternal healthcare services are examined, NGOs appear to be underutilized as a referral destination, even when many are equipped to offer normal delivery and emergency obstetric care services. Greater efficiency might be enabled by referring to alternative public and NGO facilities offering needed services which are closer in terms of road distance than Osmani Medical College, and comparable in cost. This would also ease referral pressure on Osmani Medical College which also shoulders a substantial patient load from surrounding rural areas in the District [43]. Using average figures for walking speed, this reduction in referral distance would result in a time savings of up to 25 min for one round trip in the worst-case scenario of foot travel. For example, if existing NGOs were to assume some of the current referral burden for maternal NVD, EmOC and C-section services, travel distance could be reduced by 0.4 km, 1 km, and 0.3 km respectively. This holds true for private sector referrals as well, with distances reduced by 0.6–1.1 km in the alternative scenario. The 100% evening availability of private sector services offers further potential efficiencies, especially for the working population. But for the urban poor, the costs of private sector care are often prohibitive. In this respect, strengthened referral networks involving NGOs as referral destinations hold particular promise, especially for maternity services. For emergency care services, our models suggest referral distances could be shortened by involving the private sector. However, in the absence of social protection or health insurance systems that protect the poor from impoverishing out-of-pocket medical expenditures, or of functioning regulatory mechanisms that ensure quality of care, existing referral linkages to public hospitals may be more appropriate.

### Limitations

The main limitation of this modelling exercise is that the “alternative” referral scenarios it constructs are based on reported information provided by facility owners and managers. These reported data were not verified physically, nor were actual referrals tracked or the capacity of suggested alternative facilities assessed. It is also possible that other factors might affect the practicality of the “more efficient” referral options proposed here such as geographic barriers due to variable traffic conditions, lack of adequate transport, or socio-economic and cultural barriers [21, 45]. Nevertheless, it seems reasonably safe to assume that shortest geographic distance should convert to lower travel times, especially where the difference in distance between current and alternative referral scenarios are larger. While these limitations caution against the issuance of definitive recommendations, notwithstanding they suggest substantial inefficiencies in the referral system can and should be remedied, especially for the urban poor.

It should also be noted that the analysis did not address a widespread phenomenon among public sector doctors involved in dual practice, whereby patients seen in public hospitals are referred to their personal private practice although low-cost treatment in the public system is available [12, 24, 46]. The prescription of pathological tests in private diagnostic centers where doctors may have personal interests, is an example of this, even when 90% of pathological tests and other investigations are available in public sector hospitals [12, 24, 46]. These practices fall within the remit of our concern for more evidence-informed referral practices but require further specific study and policy attention.

In response to concerns about the relevance of study findings based on 2012–2013 data, we acknowledge some NGOs may have changed, and private sector facilities may have increased. To our knowledge, however, no major policies or regulations surrounding referral practises have been implemented, and private facilities continue to locate based on economic considerations and not concerns about equitable access for the poor. In this context, we would expect that referral patterns would continue to focus on Osmani Medical College. Moreover, with the addition of new private facilities since the time of survey, we might anticipate an even higher efficiency to be achieved for alternate referral scenarios than those modelled in this paper.

Finally, as regards the generalizability of study findings beyond SCC, we acknowledge substantial variations between City Corporations (CCs) in terms of land area, population density, average household income, health facility density and range of service availability. For example, in terms of absolute numbers, Dhaka City Corporation has more NICU facilities than SCC [47]. Compared to CCs like Dhaka and Chittagong which are far larger in land area, it is likely that overall decreases in alternative referral distances would be smaller in SCC. Notwithstanding these differences, we expect referral practices in all CC settings to be primarily motivated by economic considerations and not concerns about service availability, accessibility or affordability. Further, by using distance as a measure of referral efficiency we make it easier to extrapolate the significance of our results to other settings, where differences in types of transport and traffic conditions might exist.

## Conclusions

This GIS-based modeling exercise suggests that greater efficiency in referral is possible through the use of alternate receiving facilities that provide required services that are more convenient to the urban poor in terms of proximity and working hours. However, impeding the development of a more efficient referral system is the lack of policy instruments and functional coordination bodies to enable its design and implementation. Until such mechanisms are in place, initial efforts might focus on making information about facilities, services offered, service hours and location accessible to providers at the primary level so that more appropriate referral decisions can be made. Towards this end, the development of interactive tools that utilize GIS and facility data to enable better informed decision making about referral hold particular promise [48, 49]. More informed referrals might help decrease the heavy burden on public tertiary hospitals like Osmani Medical College by redirecting cases that could be better handled at other secondary facilities, either public, private-for-profit, or NGO. At the same time, it will be important to address some of the underlying private sector incentives or motivations behind the practice of referring complex or emergent cases to Osmani to evade responsibility for potential patient deaths [12]. The development of strong first aid and emergency transport systems and life support training are important first steps in ensuring that critical patients can be moved quickly and safely to facilities where their emergency needs can be addressed [21]. In the longer term, urban health systems planning should ensure that staffed and well-equipped emergency tertiary facilities be distributed throughout the city, to lessen patient flow to Osmani Medical College, and deal with emergent cases in a timely manner. Similar actions have been undertaken in the NGO sector, with efforts to distribute maternity services closer to where the urban poor reside [50].

In addition to work with referring facilities, advocacy is needed to discourage patients from inappropriately by-passing primary and secondary health facilities, where their health concerns might be more adequately addressed [51]. In the absence of well-developed or effective referral systems in many parts of the world, it remains that a substantial number of patients access hospitals directly, without referral and without seeking prior sources of care [23].

Finally, in efforts to make referral more efficient and to ensure that referral recommendations are followed-up by patients, issues of quality and cost must be addressed [17]. Referring providers and facilities require strengthened capacities in making appropriate and timely clinical decisions, while receiving facilities must provide services of quality which are affordable to the patient [17]. Strengthening these important determinants of successful referral will require larger investments in health human resources, health systems governance and financing, and should constitute priorities for Bangladesh in implementing its new sector plan and the goal of universal health coverage.

## Data Availability

All data from the Sylhet GIS mapping study is openly accessible on the official website of the Directorate General of Health Services (DHGS), Ministry of Health and Family Welfare of the Government of Bangladesh at the following link: http://urbanhealthatlas.dghs.gov.bd/

## Abbreviations

LMICs: Low- and middle-income countries
NGO: Non-Governmental Organization
SCC: Sylhet City Corporation
GIS: Geographic Information Systems
EmOC: Emergency Obstetric Care
NVD: Normal Vaginal Delivery
C-section: Caesarian Section delivery
ICU: Intensive Care Unit
CCU: Coronary Care Unit
NICU: Neonatal Intensive Care Unit
